# Intestinal transepithelial permeability of oxytocin into the blood is dependent on the receptor for advanced glycation end products in mice

**DOI:** 10.1038/s41598-017-07949-4

**Published:** 2017-08-11

**Authors:** Haruhiro Higashida, Kazumi Furuhara, Agnes-Mikiko Yamauchi, Kisaburo Deguchi, Ai Harashima, Seiichi Munesue, Olga Lopatina, Maria Gerasimenko, Alla B. Salmina, Jia-Sheng Zhang, Hikari Kodama, Hironori Kuroda, Chiharu Tsuji, Satoshi Suto, Hiroshi Yamamoto, Yasuhiko Yamamoto

**Affiliations:** 1 Department of Basic Research on Social Recognition and Memory, Research Centre for Child Mental Development, Kanazawa University, Kanazawa, 920-8640 Japan; 2 Departments of Biochemistry and Molecular Vascular Biology, Kanazawa University Graduate School of Medical Sciences, Kanazawa, 920-8640 Japan; 3 Department of Biochemistry, Medical, Pharmaceutical and Toxicological Chemistry, Krasnoyarsk State Medical University named after Prof. V.F. Voino-Yasenetsky, Krasnoyarsk, 660022 Russia; 4 Department of Pathology, University of California, San Francisco, San Francisco, CA 94143 USA; 5 Faculty of Pharmaceutical Sciences and Center for Research and Education on Drug Discovery, Hokkaido University, Kita-12, Nishi-6, Kita-ku, Sapporo 060-0812 Japan

## Abstract

Plasma oxytocin (OT) originates from secretion from the pituitary gland into the circulation and from absorption of OT in mother’s milk into the blood via intestinal permeability. However, the molecular mechanism underlying the absorption of OT remains unclear. Here, we report that plasma OT concentrations increased within 10 min after oral delivery in postnatal day 1–7 mice. However, in Receptors for Advanced Glycation End Products (RAGE) knockout mice after postnatal day 3, an identical OT increase was not observed. In adult mice, plasma OT was also increased in a RAGE-dependent manner after oral delivery or direct administration into the intestinal tract. Mass spectrometry evaluated that OT was absorbed intact. RAGE was abundant in the intestinal epithelial cells in both suckling pups and adults. These data highlight that OT is transmitted via a receptor-mediated process with RAGE and suggest that oral OT supplementation may be advantageous in OT drug development.

## Introduction

In the United States, 12.8% of babies are born prematurely, and the number of babies worldwide that are born prematurely or at a very low body weight is approximately 15 million annually (http://www.who.int/mediacentre/factsheets/fs363/en/). These babies would not survive without extraordinary care, and they are at increased risk for health and neurodevelopmental problems^1, 2^, such as intellectual disabilities and autism spectrum disorder (ASD)^3^. The first milk and breast milk feeding thereafter are beneficial for preterm infants^4, 5^, both hormonally and nutritionally, for the development and prevention of diseases such as necrotizing enterocolitis^2, 6^. Feeding with the mother’s milk is recommended because of its beneficial effects on the infant’s intestinal microbiome^7–9^.

Breast milk contains a dynamic supply of hormones, including oxytocin (OT^4, 5, 10–15^), which is concentrated from the mother’s general circulation^16^. OT is a 9-amino-acid neuropeptide that is cleaved by intestinal peptidases but is relatively stable in gastric acid^11, 17–19^. OT in early life plays a role in the development of the social brain and in establishing social behaviour in adults^13, 20–23^. Therefore, breast milk not only nourishes beneficial microbes and provides immunity but also appears to improve well-being by improving human communication^7, 24–28^. Although OT in breast milk can be absorbed intact from the digestive tract into the blood of neonates^11, 29^, it remains to be determined whether OT is permeable after the onset of gut closure and, if permeable, whether OT absorption is a receptor-mediated process.

The intestinal epithelial cells of suckling rodents are specialized for uptake, sorting, and digestion of milk macromolecules^9^. In the proximal regions, specific IgG receptors on the luminal membranes of absorptive cells function in the selective uptake of maternal immunoglobulin and its absorption into basolateral cells^30^. Epidermal growth factor receptors have been found in membrane preparations enriched in microvillar vesicles from the small intestines of suckling rats, and specific receptor-mediated binding and endocytosis have been detected in villus cells^31, 32^. Gastric-administered insulin can lead to hypoglycaemia in suckling rats whereas no effect has been observed in 30-day-old rats^32^, thus indicating that absorption occurs in the suckling young. Furthermore, milk-borne insulin-like growth factor-1 can be absorbed into the plasma after oral gastric administration in 3-day-old piglets^33^. Some peptides can also be absorbed intact without digestion after gut closure in suckling rodents^32, 33^. Together, these results show that the intestinal epithelium forms a barrier very early in life, and macromolecules are taken up in a receptor-mediated fashion in the gastrointestinal tract^9, 34–37^.

Advanced glycation end products (AGEs) and their receptor (RAGE) are expressed in various tissues, but their physiological function is largely unknown, except in innate immunity^38^. The AGE–RAGE system has been proposed as an important pathological mechanism underlying diabetic complications such as diabetic cardiopathy, retinopathy, nephropathy, and intestinal haemorrhage^38–40^. The presence of AGE and RAGE in the small intestine has been reported in healthy animals, and their expression levels are upregulated in diabetic rodents^41, 42^. The increased expression of AGE and RAGE may contribute to diabetic gastrointestinal dysfunction, but its physiological relevance remains unclear.

Here, we tested the hypothesis that OT is freely permeable between the intestinal tract and the blood before barrier formation and that receptor-mediated permeability of OT occurs from the intestinal lumen after orogastric administration in suckling and adult mice of the C57BL/6N strain. Using RAGE knockout (KO) mice (Ref. 40; *Ager* ^−/−^), we sought to demonstrate whether such a transport mechanism depended on RAGE expressed in the intestines of infants in the early postnatal period. We also evaluated RAGE immunoreactivity in neonates and the absorption form of OT using liquid chromatography-tandem mass spectroscopy (LC-MS/MS) with a stably labelled non-radioisotope isoleucine [^13^C, ^15^N] OT.

## Results

**Figure 1:**
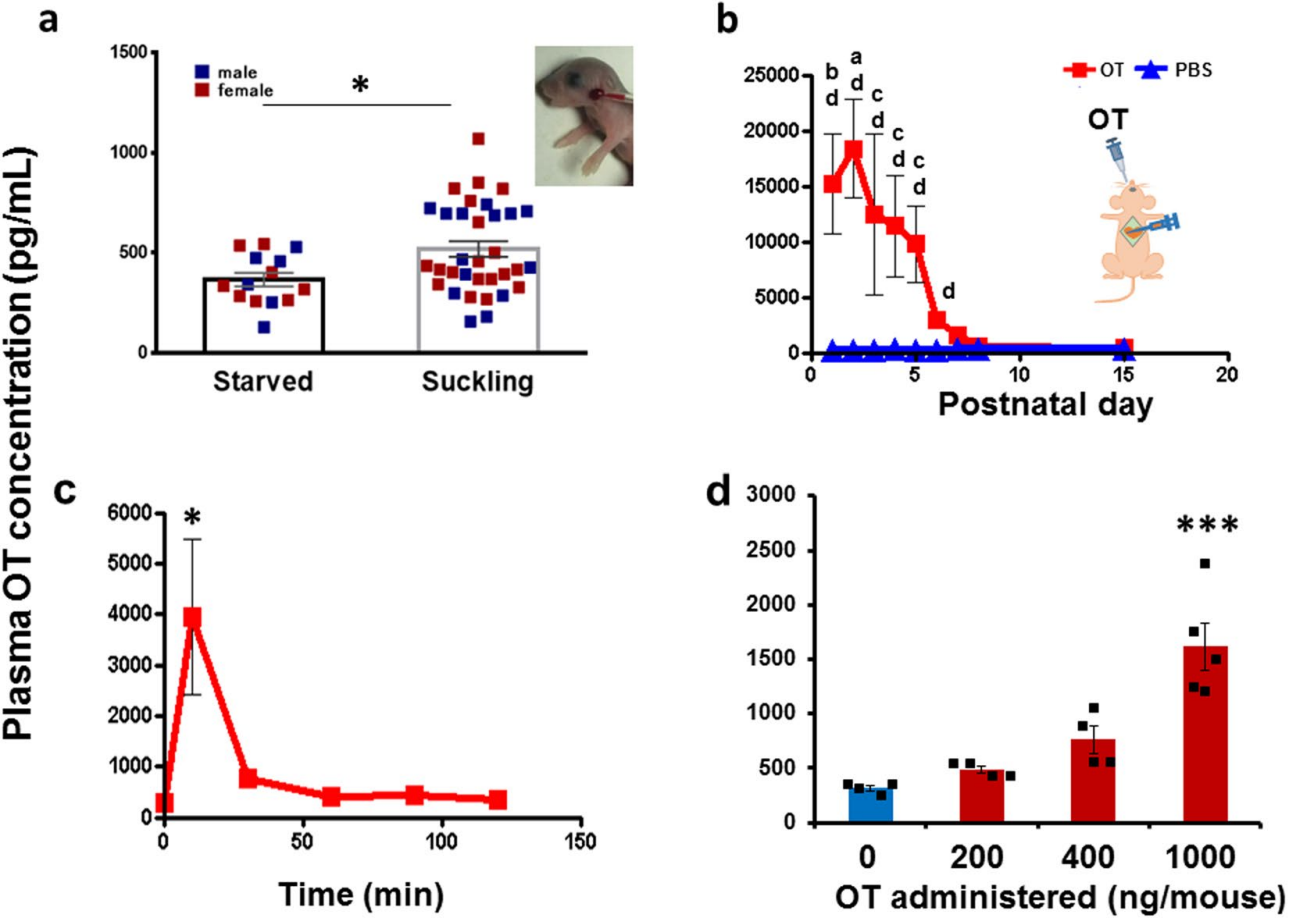
Plasma oxytocin (OT) concentrations in wild-type (C57BL/6) mice. (**a**) Plasma OT levels in PND1 male and female pups after fasting for 20 min and with or without feeding with dam’s milk for 20 min. N = 14 and 33 mice. *P < 0.05, two-tailed Student’s *t*-test. Inset shows blood collection from the facial vein. (**b**) Time course of plasma OT levels in pups 10 min after oral administration of OT (squares; 1 μg of OT/mouse; 10 μL of 100 μM OT) or PBS (triangles; 10 μL). Experiments were carried out on the indicated postnatal days. The number of mice was 3–15 for OT and 3 for saline. A two-way ANOVA revealed a significant difference between the drug treatment and saline treatment, *F*_8,90_ = 3.32, *P* < 0.002. Bonferroni’s *post hoc* tests showed ^a^ *P* < 0.001, ^b^ *P* < 0.01 and ^c^ *P* < 0.05 from the value at PND8; ^d^ *P* < 0.01 for the saline group. Inset shows the scheme of OT administration to the mice and blood collection from the heart after opening the chest. (**c**) Time course of plasma OT levels of PND6 pups measured at the indicated times after oral administration of 1 μg of OT/mouse, N = 4–5 mice. One-way ANOVA, *F*_5,37_ = 3.302, *P* < 0.0001; *P < 0.02 from the control value. (**d**) Plasma OT concentrations in PND6 pups after 10 min of oral administration of OT at the indicated concentrations. N = 3 mice. One-way ANOVA, *F*_3,24_ = 20.4, *P* < 0.0001. Bonferroni’s *post hoc* tests showed ***P < 0.001 from other values.

In suckling male and female C57BL/6N mice at PND1, the plasma OT concentration in blood samples collected from the facial vein (Fig. 1a, inset) was higher in 33 pups fed with their mother’s milk for 20 min than in pups who were fasted for 20 min (n = 14) (two-tailed Student’s *t*-test, *P* < 0.05; Fig. 1a). No clear difference in the OT level was observed between male and female pups. This result suggests that milk-borne OT is transmitted from the mother to 1-day-old pups, similar to [^3^H]OT transfer from rat mothers to neonates^11^.

### Plasma OT levels after oral delivery in suckling pups

The possibility of transport during the suckling period of development (from PND1 to PND15) was confirmed by comparing plasma OT levels at 10 min after oral delivery with 10 μL of 100 μM synthetic OT solution (1 μg/pup) or the same volume of phosphate buffered saline (PBS) to male and female pups after fasting for 30 min. The time course shown in Fig. 1b shows that plasma OT concentrations collected from the heart (Fig. 1b, inset) were markedly higher in newborn (PND1) and 2-day-old (PND2) pups than in those administered oral saline, similarly to the results of studies in suckling piglets^33^. The plasma OT concentrations after oral OT delivery remained at higher levels at PND3–5 than in the saline controls (two-way analysis of variance (ANOVA), *F*_8.90_ = 3.32, *P* < 0.002: Bonferroni’s *post hoc* tests for differences between OT and PNDs (^a^ *P* < 0.001, ^b^ *P* < 0.01, and ^c^ *P* < 0.05 from the value at PND8) and between OT and PBS (^d^ *P* < 0.01). The OT concentration dropped suddenly after PND6, although it remained higher than that in the saline control group. At PND8 and PND15, no concentration difference in plasma OT levels was observed between pups orally administered OT *vs*. those administered PBS.

The time profile of the plasma OT concentration after oral administration of OT peaked at 10 min in pups at PND6 (n = 5–8, one-way ANOVA, *F*_5,37_ = 3.302, *P* < 0.02 from the control value; Fig. 1c). The plasma OT level depended on the oral OT dose in pups at PND6 (one-way ANOVA, *F*_3,24_ *= *20.35, *P* < 0.0001; Fig. 1d).

**Figure 2:**
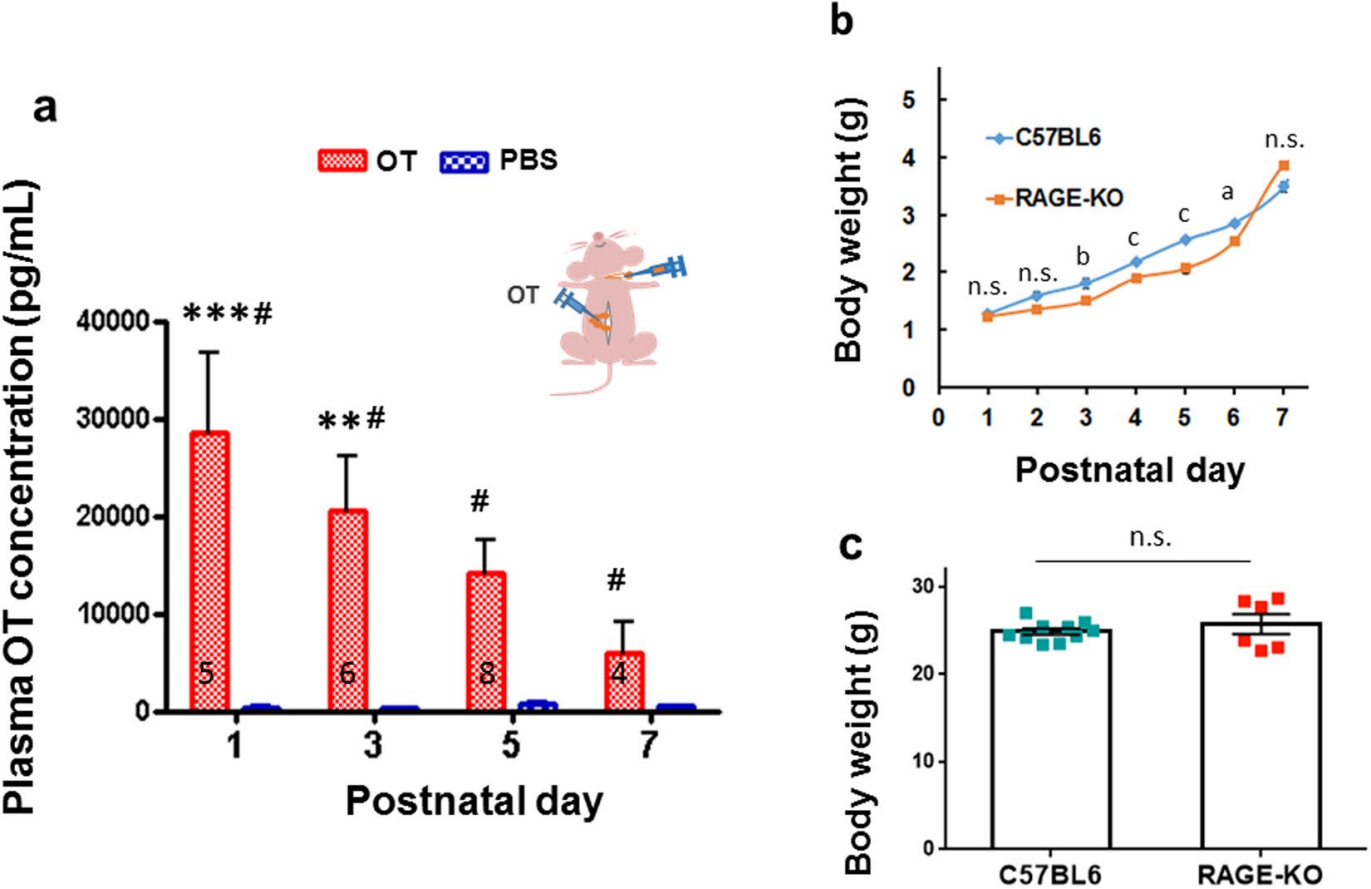
Plasma OT levels in pups after intestinal delivery of OT. (**a**) Blood samples were collected from the carotid artery after cutting the neck of male and female pups at PND1, 3, 5, and 7, 10 min after the administration of OT (Red; 100 μM × 5 μL, 0.5 μg/mouse) or the same volume of PBS (Blue) into the upper small intestinal tract (inset). Two-way ANOVA, *F*_3,40_ = 3.14, *P* < 0.05. Bonferroni’s *post hoc* tests showed significant differences between OT and saline (^#^ *P* < 0.001); OT and PNDs, ***P < 0.001, **P < 0.01 from values at PND7. The number of experiments is shown. (**b**) Weight curve during the 7 days after birth for wild-type (blue diamonds, n = 104) and *Ager* ^−/−^ (RAGE KO; orange squares, n = 78) male and female pups. A two-way ANOVA revealed significant differences between body weights of wild-type and KO mice for *Genotypes* (*F*_1,6_ = 11.81 *P* < 0.007), *PNDs* (*F*_1,6_ = 142.95, *P* < 0.0001) and interaction between *Genotypes x PNDs* (*F*_2,278_ = 5.10, *P* < 0.0001). Bonferroni’s *post hoc* tests showed 0.493 (not significant (n.s.)), 0.154 (n.s.), 0.003 (b), 0.00005 (c), 0.00002 (c), 0.002 (a), 0.057 (n.s.) for *P* values at PND1-7, respectively. (**c**) Body weights of adult males of 11 week-old mice (n = 10 and 6 for wild-type and RAGE KO mice, respectively); Two-tailed Student *t*-test, *P* = 0.395, not significant.

To confirm that the above increase after oral administration was primarily owing to permeability from the intestine, 5 μL of 100 μM OT (0.5 μg/pup) was applied directly into the small intestine, bypassing the mouth, buccal mucosal membrane, and/or upper parts of the digestive tract, including the oesophagus and stomach. The plasma OT in blood samples collected from the neck (Fig. 2a, inset) reached a peak at 10 min and decreased at 60 min and 120 min after intestinal application (Supplementary Fig. 1). The mean plasma OT concentration in groups of 4–8 male and female pups at PND1–7 was significantly higher in the OT group than the PBS control group (two-way ANOVA, *F*_3,40_ = 3.14, *P* < 0.05; Bonferroni’s *post hoc* tests for differences between OT and PBS (^#^ *P* < 0.001) and OT and PNDs; Fig. 2a, red vs. blue bars). The plasma OT level was higher at PND1 and PND3 than at PND7 in the group injected with OT (***P < 0.001 and **P < 0.01; Fig. 2a).

The plasma OT concentration at 10 min after intestinal OT injection decreased by approximately 4.6-fold from PND1 to PND7 (Fig. 2a). However, this decrease may not simply reflect a decrease in the total amount of OT absorption and may also be related to the blood volume increase associated with body weight gain during development. Because it is difficult to measure the total blood volume, we estimated it from the body weight. We observed a 2.7-fold body weight gain, from 1.3 ± 0.03 g at PND1 (n = 26 male and female unique individual neonates; Fig. 2b, blue line) to 3.5 ± 0.08 g at PND7 (n = 57 pups). Considering this body weight gain and the linear blood volume increases with grams in neonates^43^, it is likely that total OT absorption slightly decreased, probably owing to intestinal barrier and/or digestive development. Altogether, these data in wild-type mice suggest that OT passes into the blood across the digestive tract, including the intestinal mucus and epithelial cells, and crosses the intestinal epithelial barrier^34, 37, 44^.

On the other hand, a 3.0-fold increase in body weight was observed during PND1–7 among male and female RAGE KO pups with C57BL/6 background (*Ager* ^−/−^) (Fig. 2b, red line). A two-way ANOVA revealed significant differences between body weights of wild-type and KO mice for *Genotypes* (*F*_1,6_ = 11.81, *P* < 0.007), *PNDs* (*F*_1,6_ = 142.95, *P* < 0.0001) and interaction between *Genotypes x PNDs* (*F*_2,278_ = 5.10, *P* < 0.0001). Bonferroni’s *post hoc* tests showed significant differences, with *P* < 0.01 for PND3, *P* < 0.0001 for PND4 and PND5, and *P* < 0.001 for PND6 (Fig. 2b), suggesting that RAGE deletion elicited a developmental delay of 11–19% in body weight during PND3–6. However, no impairment in the average body weight of 11-week-old male adults was seen (Fig. 2c): 24.9 ± 0.4 g (n = 10 wild-type mice) *vs*. 25.7 ± 1.3 g (n = 6 KO mice); two-tailed *t*-test, *P* = 0.395. Interestingly, in terms of body weight gain, there was a significant difference between wild-type and KO mice. This prompted us to investigate RAGE-dependent absorption in RAGE KO mice.

### Plasma OT levels in RAGE KO pups

**Figure 3:**
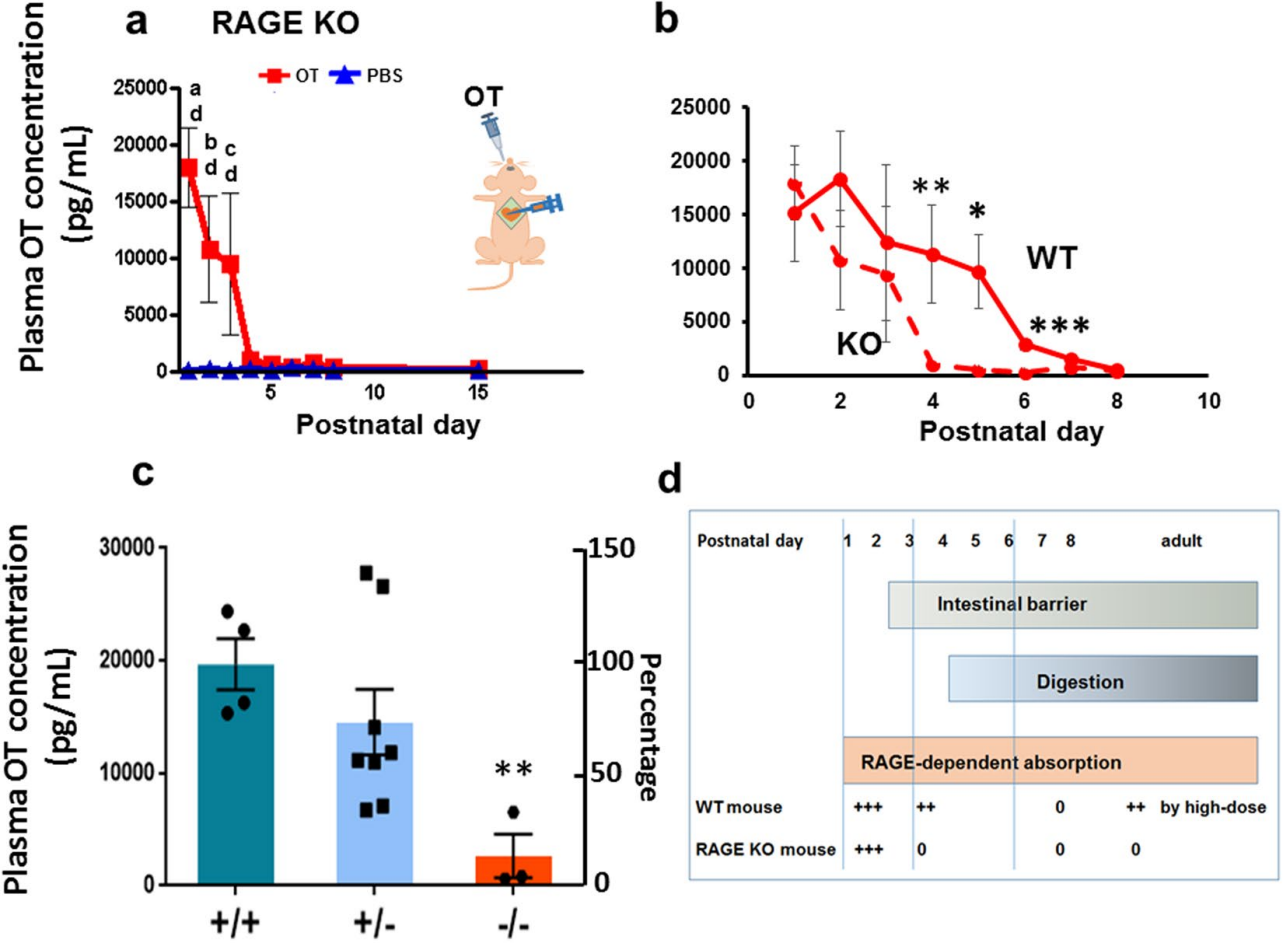
Plasma OT concentrations in *Ager* ^−/−^ (RAGE KO) mice. (**a**) Time course of plasma OT levels in pups 10 min after the oral administration of 1 μg of OT/mouse (squares; 100 μM × 10 μL) or saline (triangles; 10 μL). Blood samples were collected from the hearts of male and female pups at the indicated postnatal days. Inset indicates oral OT delivery and blood collection from the heart. The number of experiments was 3–15 for OT and 3 for saline. Two-way ANOVA, *F*_8,90_ = 5.16, *P* < 0.001. Bonferroni’s *post hoc* tests showed significant differences between OT and PBS (^d^ *P* < 0.001) or OT and PNDs, as indicated (^a^ *P* < 0.001, ^b^ *P* < 0.01, ^c^ *P* < 0.05 from PND8). (**b**) Plasma OT levels in wild-type and RAGE KO pups 10 min after oral administration of OT. The data are re-plotted from Figs 1b and 3a. Two-way ANOVA, *F*_1,126_ = 4.73, *P* < 0.05. Bonferroni’s *post hoc* tests showed significant differences between wild-type and RAGE KO mice at *P < 0.05, **P < 0.01, and ***P < 0.001. (**c**) Plasma OT concentrations in *Ager* ^+/+^ (n = 4), *Ager* ^+/−^ (n = 8) and *Ager* ^−/−^ (n = 3) pups of the litter mates of 3 dams at PND5 after oral OT administration as in **a**. One-way ANOVA, *F*_2,22_ = 6.851, *P* < 0.01. Bonferroni’s *post hoc* tests revealed a difference between *Ager* ^+/+^ and *Ager* ^−/−^ mice, *P* < 0.01. (**d**) The scheme illustrates the relationship between plasma OT increases and barrier formation, digestion, and RAGE-dependent absorption after OT delivery during development. 0, + , +  + , +  +  + indicates from little to no permeability to the highest permeability and resulting plasma OT levels.

Using RAGE KO mice, we next examined OT increases beyond the basal level in the blood after oral OT administration (100 μM × 10 μL; 1 μg/pup). The plasma OT concentrations in cardiac samples (Fig. 3a, inset) in *Ager* ^−/−^ male and female pups (n = 38) were significantly increased from PND1–3 in the oral OT group than the saline group (Fig. 3a; two-way ANOVA, *F*_8,90_ = 5.16, *P* < 0.001). Bonferroni’s *post hoc* tests for differences between OT and PBS (*P* < 0.001) and between OT and PNDs (^a^ *P* < 0.001, ^b^ *P* < 0.01, and ^c^ *P* < 0.05 from PND8, respectively). The OT concentration dropped suddenly at PND4 in RAGE KO pups, and there were no subsequent differences in OT concentrations between pups orally administered OT or PBS.

The elevated levels of OT at PND1–3 were similar between the two genotypes (Fig. 3b). However, interestingly, from PND4 to PND6, significantly lower plasma OT concentrations were observed in RAGE KO mice with orally administered OT than in wild-type mice (two-way ANOVA, *F*_1,126_ = 4.73, *P* < 0.05). Bonferroni’s *post hoc* tests showed significant differences between wild-type and RAGE KO mice with *P < 0.05 (for PND5), **P < 0.01 (PND4), and ***P < 0.001 (PND6). This RAGE dependency was lost after PND7.

To test gene-dosage effects on OT absorption, we examined plasma OT concentrations in pups of three genotypes in the same litter at PND5, using the same method as in Fig. 3a. The plasma OT level in *Ager* ^+/+^ pups was as high as that in *Ager* ^+/−^ pups and significantly higher than that in *Ager* ^−/−^ pups, as expected (Fig. 3c; one-way ANOVA, *F*_2,22_ = 6.851, *P* < 0.01; Bonferroni’s *post hoc* test for the difference between *Ager* ^+/+^ and *Ager* ^−/−^, *P* < 0.01).

All these results show that the higher OT concentrations in neonates to 3-day-old pups of both genotypes were likely to be owing to non-selective transmission, probably resulting from leakage before closure of the intestinal barrier (as indicated by +++ in Fig. 3d; ref. 34, 37). Then, after PND4, permeability decreased, owing to cessation of non-selective transmission, although selective transmission remained after closure of the epithelium and barrier formation. Alternatively, a small nutritional abnormality in KO mice during PND3–6 may be owing to disturbance in the RAGE-dependent absorption of general nutrients. Thus, OT may permeate the intestine through a RAGE-dependent receptor mechanism (Fig. 3d).

### OT transport from isolated intestines of suckling pups

**Figure 4:**
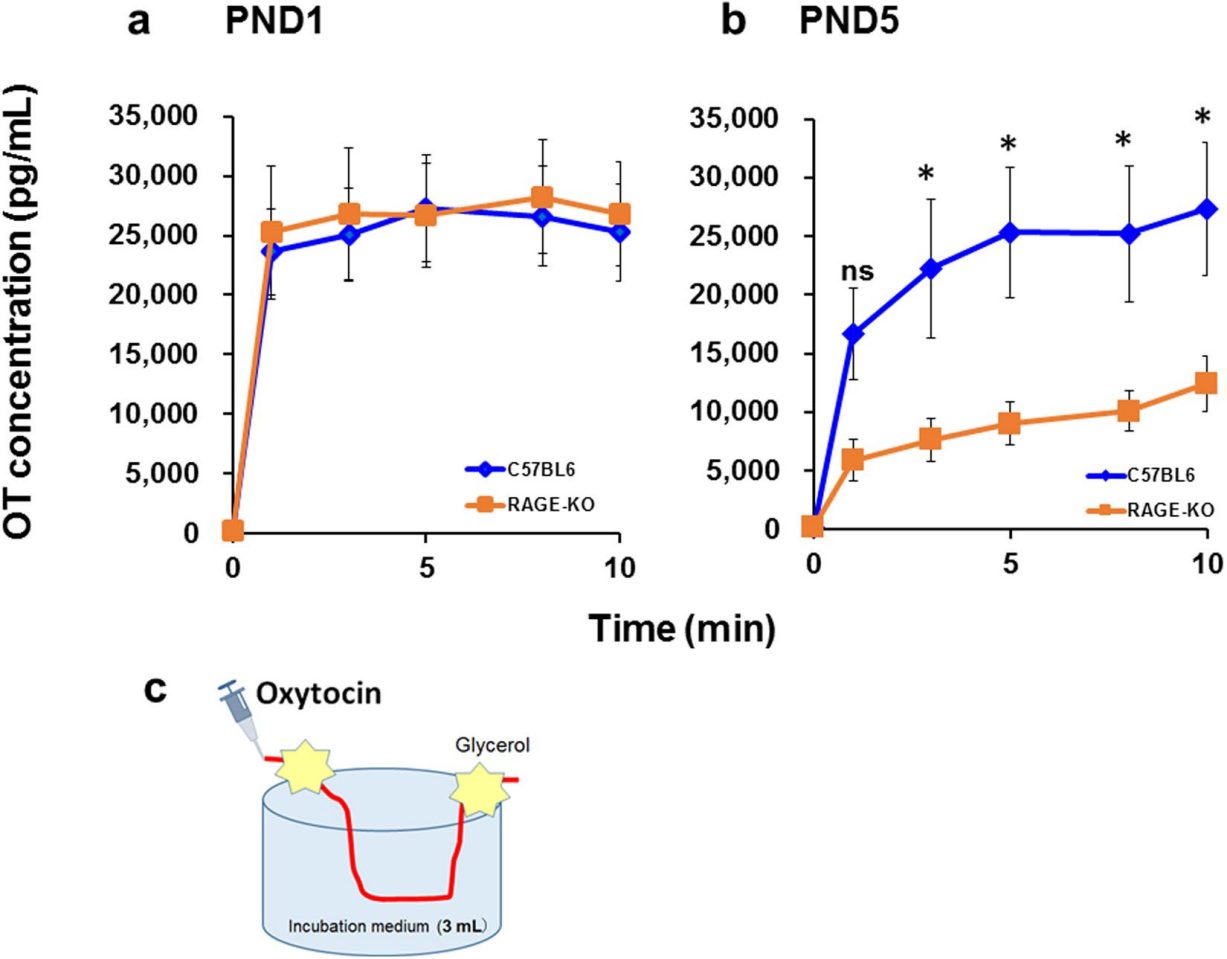
OT concentrations in incubation medium from isolated intestines. Time course of OT concentrations in incubation medium (3 mL) containing a segment of the intestine isolated from PND1 (**a**) or PND5 (**b**) wild-type (C57BL6, blue diamonds) and *Ager* ^−/−^ (RAGE KO; orange squares) pups. Fifty microlitres of 10 μM OT was injected at the distal end after ligation. Fifteen microlitres of incubation medium was collected at the indicated time after injection of OT. In (**b**), two-way ANOVA for genotype effect, *F*_1,84_ = 31.02, *P* < 0.0001 and for time effect, *F*_5,84_ = 7.41, *P* < 0.0001. Bonferroni’s *post hoc* tests revealed significant differences between wild-type and KO mice at ^*^ *P* < 0.05. (**c**) The scheme illustrates the incubation of the isolated intestines.

Non-selective and selective transmission of OT from the intestinal mucosa was examined (Fig. 4), using isolated segments of the upper intestine. Intestinal segments (4 cm in length, from 1–2 cm below the stomach) were incubated in 3 ml of saline solution in a chamber at room temperature (Fig. 4c). OT (10 μM × 50 μL; 0.5 μg/intestine) was injected directly into the mucosal lumen at the distal cutting edge, which was isolated from the incubated segments with glycerol. OT concentrations in the incubation medium were monitored for 10 min. Figure 4a shows that high OT levels were achieved soon after the start of incubation, and there were no differences between genotypes in neonates (PND1). In 5-day-old pups (PND5), the OT concentrations gradually increased after smaller instantaneous increases in both wild-type (C57BL6) and *Ager* ^−/−^ (RAGE KO) pups (Fig. 4b). A two-way ANOVA for interaction between *Genotypes* × *Time* revealed no significance (*F*_5,84_ = 1.39, *P* = 0.2346), but a significant difference was found for the *Genotype* effect (*F*_1,84_ = 31.02, *P* < 0.0001) and the *Time* effect (*F*_5,84_ = 7.41, *P* < 0.0001). Bonferroni’s *post hoc* tests showed significant differences with *P* < 0.05 between wild-type and RAGE KO mice during 3–10 min (Fig. 4b), with respect to OT concentrations in the incubation medium.

It is of interest to note that transluminal absorption in the wild-type mice at 10 min was not different *in vitro* at P1 and P5 (Fig. 4) whereas there was a decrease in transluminal absorption sampled at 10 min for each age (Fig. 2a). This is probably owing to the denominator (body volume) being kept constant; during *in vivo* experiments, the denominator (pup “volume”) was not held constant because the pups were larger at older ages, resulting in more dilute recovered OT.

The leakage level of the isolated intestines was examined by fluorescein isothiocyanate (FITC)-dextran permeability^45^. Leakage levels were similar between the two genotypes at the two different PNDs (n = 3, *P* = 0.67 for PND1 and *P* = 0.59 for PND6; Table 1). This suggests that the isolated intestines of both genotypes were functional, without much damage, although transepithelial electrical resistance should be measured to determine epithelial barrier formation^45^.

**Table 1:** Intestinal permeability measured by FITC-dextran.

	Permeability (units/min)	
	PND1	PND6
Wild-type	13.0 ± 0.9^a^	17.1 ± 0.3^c^
*Ager* ^−/−^	13.4 ± 0.4^b^	16.9 ± 0.3^d^

To assess intestinal tissue permeability using a FITC-dextran assay, 4-kDa FITC-dextran was injected into the mucosal side of the isolated intestine as in Fig. 4. Samples were collected at the beginning of incubation and after 3 min. The fluorescence of the samples was quantified by a spectrofluorophotometer (Model RF6000, Shimadzu, Kyoto, Japan): the excitation wavelength was 485 nm, and the emission wavelength was 538 nm. The result obtained at 0 min (arbitrary units) was subtracted from the result obtained at 3 min, and this difference was then divided by 3 min. N = 3 for each genotype. Two-tailed Student *t*-test: *P* = 0.67 between a and b; *P* = 0.59 between c and d; *P* < 0.01 between a and c; *P* < 0.002 between b and d, respectively.

### Plasma OT levels in adult mice

**Figure 5:**
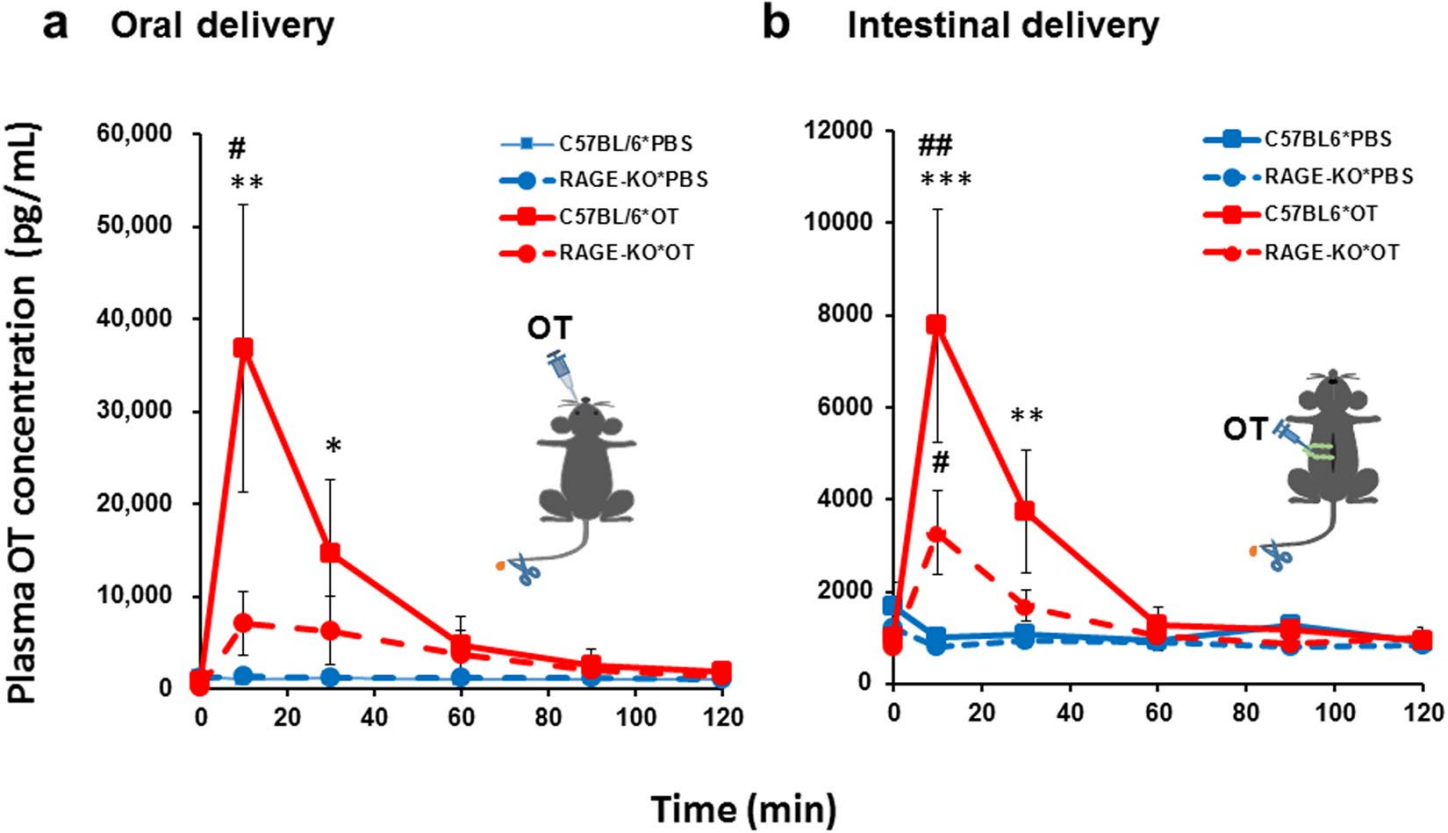
Plasma OT levels in adult male mice after oral or intestinal delivery of OT and PBS. Blood samples were collected at the indicated time from the tail after administration of OT (10  μg/mouse) into the mouth (100 μM × 100 μL, (**a**) or the upper intestinal tract (100 μM × 100 μL, (**b**). Wild-type (WT; N = 3 for (**a)** and (**b**) or *Ager* ^−/−^ (RAGE KO; N = 4 for (**a)** and **b**) mice of PND56-65 were anesthetized, and the tails were cut repeatedly for the collection of 30 μL of blood (Insets). (**a**) Two-way ANOVA, *F*_1,48_ = 4.66, *P* < 0.05. Bonferroni’s *post hoc* tests showed significant differences between the two genotypes (^#^ *P* < 0.01). Significantly different from the control value at *P < 0.05 and **P < 0.01, respectively. (**b**) A two-way ANOVA for interaction between *Genotypes* and *Time* showed *F*_15,168 = _4.25, *P* < 0.0001. A two-way ANOVA for *Genotype* × *Treatment* effect revealed significances, *F*_3,168_ = 8.58, *P* < 0.0001 and for the *Time* effect, *F*_5,168_ = 7.58, *P* < 0.0001. Bonferroni’s *post hoc* tests show significant differences between PBS and OT in wild-type mice at ***P < 0.001 and *P < 0.05; between saline and OT in RAGE KO mice at ^#^ *P* < 0.05; and OT levels between wild-type and KO mice at ^##^ *P* < 0.001.

In adult male mice (8 weeks old), when oral OT was administered at the same dose used for neonates (1 μg/mouse), as in Fig. 1b, no increase in the plasma OT concentration was observed (data not shown). To examine whether permeability exists in the adult intestine after barrier formation, a 10-fold higher volume of OT (100 μM × 100 μL) was used for oral delivery (10 μg/mouse). Blood samples were collected from anesthetized mice using a tail snip procedure, and OT concentrations were measured (Fig. 5a, inset). A significant increase in plasma OT was observed at 10 min after administration in wild-type (C57BL/6) mice whereas the levels in RAGE KO mice were significantly lower (Fig. 5a; two-way ANOVA for genotype differences, *F*_1,48_ = 4.66, *P* < 0.05). Bonferroni’s *post hoc* tests showed significant differences in OT concentrations between the two genotypes after oral OT administration (^#^ *P* < 0.01). A two-way ANOVA (*F*_5,48_ = 4.53, *P* < 0.002) indicated a difference between *Time and Genotypes*, which was significantly different from the control value with *P < 0.05 at 30 min and **P < 0.01 at 10 min after OT administration in wild-type mice. The basal OT level before administration of OT or after oral saline administration was similarly low in both genotypes (Fig. 5a, blue line), suggesting that anesthetization of the mice did not influence the OT levels.

The OT concentrations were determined in plasma obtained from tails after direct administration of OT into the intestine (100 μM × 100 μL; 10 μg/intestine), which is partially exposed to the abdominal cavity during injection, to avoid contamination from other tissues (Fig. 5b, inset). The OT level significantly increased after 10 min of direct OT delivery into the mucosa of the upper intestine; this level was very low in *Ager* ^−/−^ mice (Fig. 5b; two-way ANOVA for interaction between *Genotypes* and *Time*, *F*_15,168_ = 4.25, *P* < 0.0001). A two-way ANOVA for the *Genotype* × *Treatment* effect revealed significant differences, *F*_3,168_ = 8.58, *P* < 0.0001 and for the *Time* effect, *F*_5,168_ = 7.58, *P* < 0.0001. Bonferroni’s *post hoc* tests show significant differences between saline and OT in wild-type mice with ***P < 0.001 and *P < 0.05, between saline and OT in RAGE KO mice with ^#^ *P* < 0.05, and OT levels between wild-type and KO mice with ^##^ *P* < 0.001.

The plasma OT concentration increased in a linear fashion as a function of the dose applied directly to the intestine (Supplementary Fig. 2). In addition, the same volume of saline delivered into the intestine did not increase the plasma OT level.

### Vasopressin levels in the plasma after oral delivery and transport from isolated intestines

**Figure 6:**
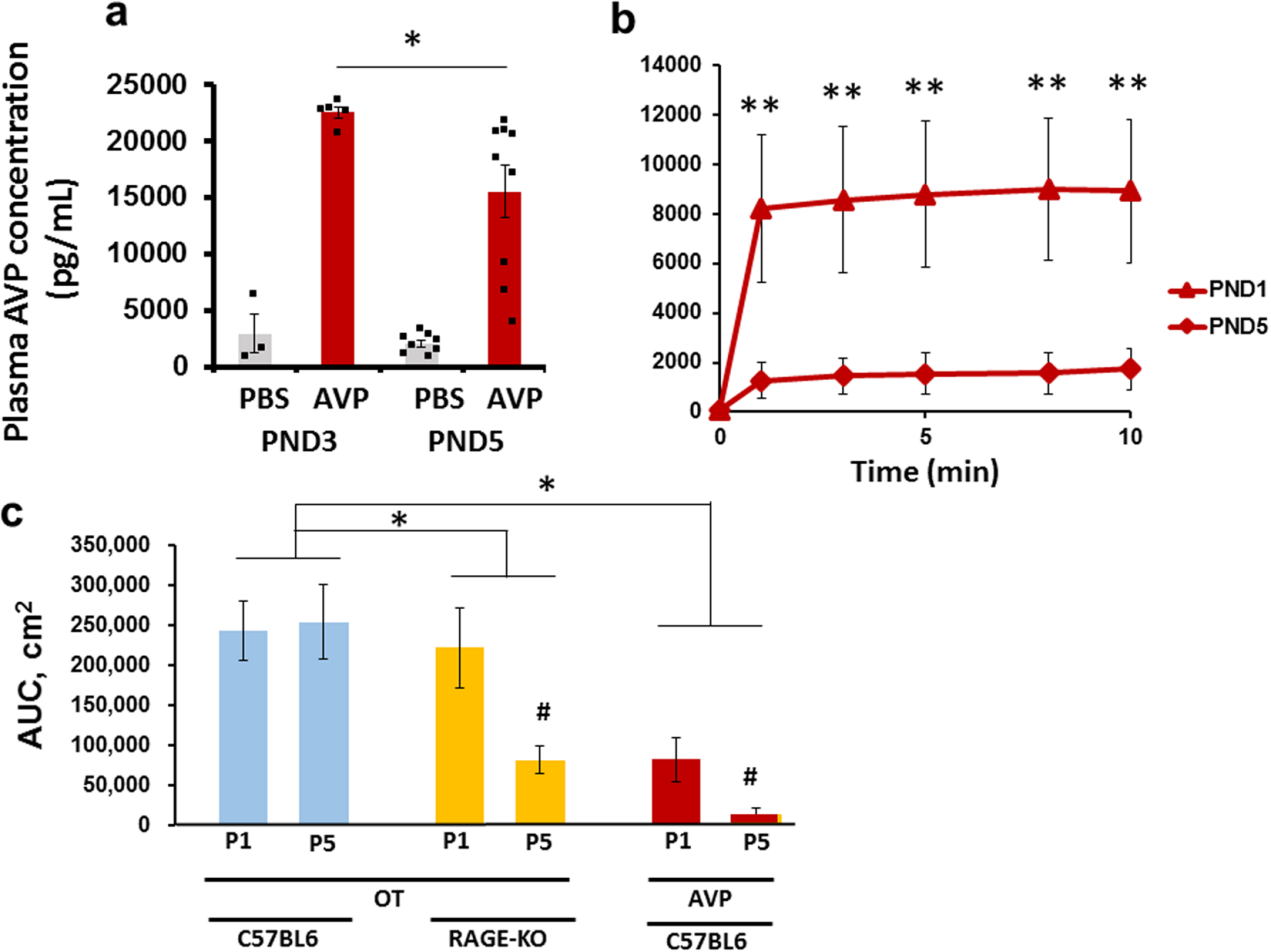
Plasma vasopressin concentrations in wild-type mice. (**a**) Plasma arginine vasopressin (AVP) levels in PND3 and 5 pups 10 min after oral administration of AVP (orange bars; 1 μg of AVP/mouse) or PBS (blue bars; 10 μL). N = 5 each. A two-way ANOVA revealed a significant difference between the two *Treatments (PBS/AVP)*, *F*_1,18_ = 5.21, *P* < 0.05; and for the *Age* effect, *F*_1,18_ = 91.26, *P* < 0.0001. Bonferroni’s *post hoc* tests revealed P < 0.05 between AVP levels at PND3 and 5. (**b**) Time course of AVP concentrations in incubation medium (3 mL) containing a segment of the intestine isolated from PND1 (triangles) or PND5 (diamonds) wild-type pups. Five microlitres of 100 μM AVP was injected at the proximal end after ligation. Fifteen microlitres of incubation medium was collected at the indicated time after injection. A two-way ANOVA revealed a significant difference for the *Age* effect *F*_1,40_ = 25.95, *P* < 0.0001. Bonferroni’s *post hoc* tests revealed *P* < 0.01 between AVP levels at PND1 and 5. (**c**) The area under the curve (AUC) calculated from the plots in Figs 4a,b and 6b. Note that the AUC values of OT in wild-type pups at PND1 and PND5 and KO mice at PND1 were similar, while the AUC value of OT in PND5 KO pups was significantly lower. A two-way ANOVA showed a significant difference between the *G*enotypes, *F*_1,16_ = 6.37, ^#^ *P* < 0.05. The AUC values of AVP in wild-type pups at PND1 and 5 were lower than those of OT in wild-type pups. A two-way ANOVA revealed a significant difference for the *Treatment effect, F*_1,16_ = 39.92, *P < 0.05

To study the specificity of RAGE-dependent transport of OT, we investigated the intestinal absorption of another nonapeptide, arginine vasopressin (AVP^46^) by oral delivery and in the isolated intestines. Transport during the suckling period of development (PND3 and PND5) was confirmed by comparing plasma AVP levels at 10 min after oral delivery of 10 μL of synthetic AVP solution (100 μM; 1 μg/pup) or the same volume of PBS to pups after fasting for 30 min. Plasma AVP concentrations were markedly higher at both PND3 and PND5 in pups administered AVP than in those administered oral saline (Fig. 6a). Two-way ANOVA was used to analyse the interaction between *Treatment (PBS/AVP)* × *Age, F*_1,18_ = 3.14, *P* = 0.0933; the *Treatment (PBS/AVP) effect, F*_1,18_ = 5.21, *P* < 0.05; and the *Age effect*, *F*_1,18_ = 91.26, *P* < 0.0001. Bonferroni’s *post hoc* tests were used to determine differences in AVP levels between PND3 and PND5, *P < 0.05. It is worth noting that the pup size denominator problem was also present in the above experiment. However, if we roughly estimate AVP recovery, it is approximately 4% at PND3 (22 ng was recovered from the effective dose of 555 ng (1 μg/1.8 g pup)) and also 4% at PND5 (16 ng was recovered from the effective dose of 400 ng (1 μg/2.5 g pup)).

Transmission of AVP from the intestinal mucosa was examined using the same paradigm as that in Fig. 4a. AVP was injected directly into the mucosal lumen (5 μL of 100 μM AVP), and AVP concentrations in the incubation medium were monitored (Fig. 6b). Relatively high AVP levels were achieved soon after the start of incubation in neonates (PND1). In 5-day-old pups (PND5), the AVP concentration was much lower. Two-way ANOVA was used to analyse differences in interaction between *Time* × *Age, F*_4,40_ = 0.15, *P* = 0.9616; the *Time* effect, *F*_4,40_ = 0.27, *P* = 0.8951; and the *Age* effect, *F*_1,40_ = 25.95, *P* < 0.0001. Bonferroni’s *post hoc* tests showed differences in AVP levels between PND1 and PND5, **P < 0.01 (Fig. 6b).

To estimate the amount of released AVP, we calculated the area under the curve (AUC). The AUC values of AVP were significantly lower than those of OT (Fig. 6c). Two-way ANOVA showed a significant difference between *Genotypes* (*F*_1,16_ = 6.37, *P < 0.05.) and *Treatment effect* (*F*_1,16_ = 39.92, *P* < 0.05). In addition, there was a significant decrease after development for OT in RAGE-KO pups and AVP in wild-type pups for both (^#^ *P* < 0.05 for PND1). This suggests that AVP is sensitive to barrier formation, although it is necessary to measure it in KO mice.

### Determination of OT by mass spectroscopy

Next, we examined whether increased OT after oral administration was owing to the permeability of exogenous OT across the intestinal barrier in an undigested form. The presence of the intact form of OT in the plasma was measured on the basis of the molecular weight, which was identified using LC-MS/MS^47, 48^.

To distinguish exogenous OT from endogenous OT in the plasma, we first synthesised two non-radioactive, stably labelled isoleucine [Ile ^13^C, ^15^N] oxytocin (designated as [Ile ^13^C, ^15^N]OT) and [^13^C, ^15^N] proline and [^13^C, ^15^N] leucine [Pro&Leu^13^C, ^15^N]OT with molecular mass numbers of 1014 and 1021, respectively. Standard (STD) samples of [Ile ^13^C, ^15^N]OT (100 ng/mL) for administration and [Pro&Leu^13^C, ^15^N]OT (100 ng/mL) for the internal standard were prepared by dilution of their stock solutions with 0.1% trifluroacetic acid as necessary.

**Figure 7:**
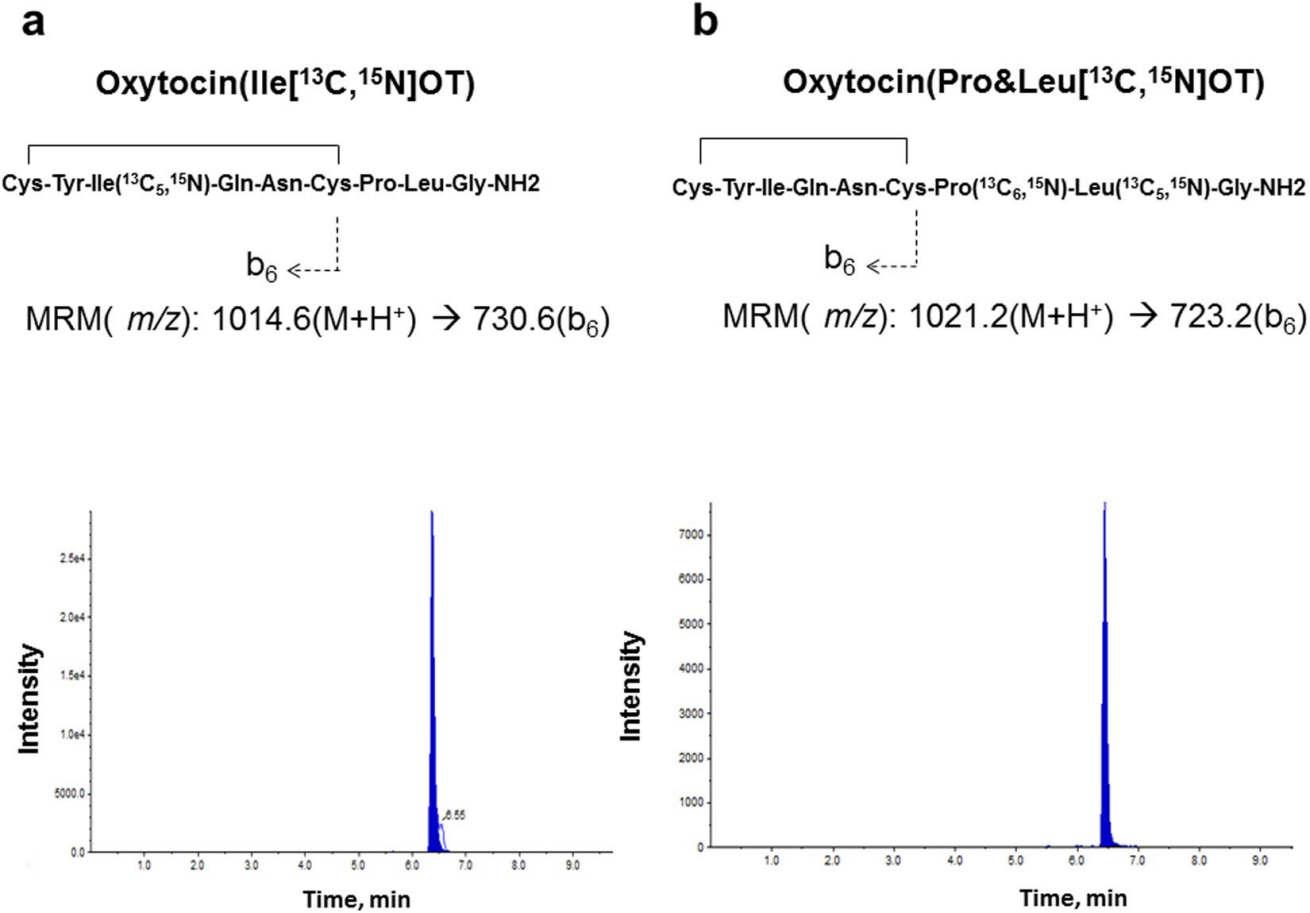
MS/MS fragmentation and MRM detection of the oxytocins Ile[^13^C, ^15^N]OT and Pro&Leu[^13^C, ^15^N]OT. The multiple-reaction monitoring MRM transitions of the two stable isotope-labelled OTs used in this study are shown. These parent ions [M + H^+^] (*m/z* 1014.6 and *m/z* 1021.2) produce the fragment (daughter) b6 ions (*m/z* 730.6 and *m/z* 723.2) after collision with N_2_ gas in the collision cell, and both b6 ions are then selectively detected. The representative MRM chromatograms (left and right) correspond to Ile[^13^C, ^15^N]OT and Pro&Leu[^13^C, ^15^N]OT in one of the four mouse plasma samples. [Pro&Leu^13^C, ^15^N]OT (10 ng/mL) was spiked as an internal standard (I-STD) into all samples.

Plasma samples were collected from the heart of anesthetized wild-type PND3 pups at 10 min after oral administration of [Ile ^13^C, ^15^N]OT (100 µM × 10 μL). The samples were fractionated with 1 N salt, and subjected to LC-MS/MS after sample preparation by the deproteination procedure. Figure 7 illustrates two representative traces of multiple reaction monitoring (MRM) chromatograms ([M + H^+^] (*m/z* 1014.6) → b6 ion (*m/z* 730.6) for the administered [Ile ^13^C, ^15^N]OT; [M + H^+^] (*m/z* 1021.2) → b6 ion (*m/z* 723.2) for the internal STD [Pro&Leu^13^C, ^15^N]OT)^47^. The left MRM chromatogram indicates the presence of full-length stably labelled [Ile ^13^C, ^15^N]OT in the blood owing to intestinal absorption after oral administration in suckling (PND3) pups (n = 4). The right MRM chromatogram of Fig. 7 corresponds to the internal STD [Pro&Leu^13^C, ^15^N]OT spiked into the plasma.

### Intestinal staining with anti-RAGE antibody

**Figure 8:**
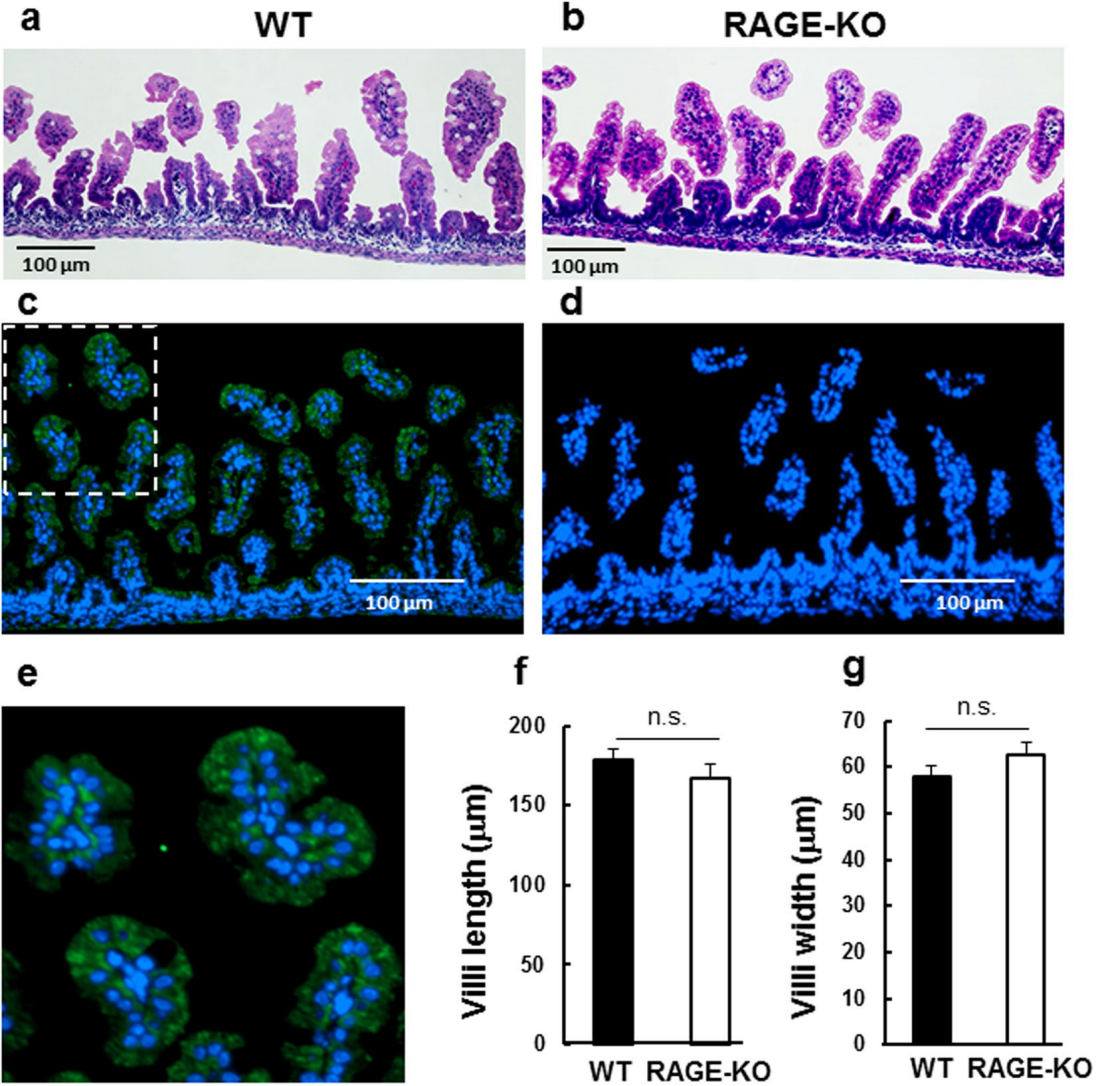
RAGE in the intestine. (**a** and **b**) Small intestines from 3-day-old wild-type (WT) and *Ager* ^−/−^ (RAGE KO) mice were isolated, fixed with 4% paraformaldehyde, and paraffin embedded. Sections (4 μm) were stained with haematoxylin and eosin. (**c** and **d**) Sections of the intestines of PND3 WT (**c**) and RAGE KO (**d**) mice were immunostained with an anti-RAGE antibody (green); the nuclei were stained with DAPI. Bar = 100 μm. Co-staining of RAGE with DAPI indicates that RAGE is present in the intestinal villi in an enlarged image (**e**). (**f** and **g**) Morphological parameters such as villus length and villus width were measured from images with haematoxylin and eosin staining. Note that there were no significant differences in these parameters between the two groups N = 3.

Expression of RAGE in the intestine has been demonstrated in adult humans and rodents^42^, but not in human infants and suckling mice. Therefore, we performed immunohistochemistry using anti-RAGE antibody (Fig. 8). Figure 8c and e illustrates RAGE immunopositive staining in the epithelial cells of the villi of PND3 pups. No identical staining was detected in *Ager* ^−/−^ pups (Fig. 8d). RAGE expression was also detected in E18.5 embryos (Supplementary Fig. 3). After extensive washing of the intestine, this staining remained unchanged, thereby suggesting that this method is likely to detect the membrane-bound form of RAGE as a component of intestinal epithelial cells. Finally, we confirmed no apparent structural abnormalities in the villi of *Ager* ^−/−^ pups by analysis of hematoxylin and eosin stained intestine tissue (Fig. 8a,b,f and g): two-tailed Student’s *t*-test between wild-type and RAGE KO mice, *P* = 0.34 for villi length and *P* = 0.18 for villi width (n = 30 counts from 3 mice).

## Discussion

Our results provide the first reported evidence of RAGE-mediated transport of OT from the intestinal tract into the blood circulation across the intestinal barrier in mice. This intestinal permeability showed an apparent difference, on the basis of plasma OT concentrations, between wild-type and RAGE KO pups at PND4–6 after oral administration of OT. This capacity was also demonstrated in adults when we used a 10-fold concentrated OT solution. Although it is known that OT is absorbed in neonates and infants^11^, our results showed that increased levels of plasma OT in both wild-type and RAGE KO mice at approximately 1–3 days after birth were equally high, probably because of nonspecific bulk absorption (Fig. 3d). However, our experiments using incubated segments of intestine provided direct evidence for the transportation of OT from the intestinal lumen into the incubation medium.

Primarily adult RAGE KO mice have been used to study various metabolic diseases^38–42^. However, KO mice do not usually display any apparent developmental impairments^40^. In the current study, after maturing, the body weight of the two genotypes (wild-type and RAGE KO) was identical. The apparent intestinal structure of the villi at microscopic level of the two types of mice was statistically no different (Fig. 8). Intestinal permeability has been shown to have mouse strain heterogeneity^45^. In this study, we used the same C57BL/6 background in both wild-type and KO mice. The leakage levels were nearly identical between the two types, and the intestinal barrier appeared to form at the expected developmental stage (Figs 4 and 6 and Table 1). These data clearly suggest that the *Ager* gene deletion resulted in modest changes in nutritional malabsorption and abnormal development, but specifically disrupted OT absorption. Therefore, this is the first report of RAGE-dependent intestinal absorption of OT at the molecular level, which we identified by comparing wild-type and *Ager* knockout mice.

OT absorption through the intestine was more difficult to detect in adults. The reasons for this difference with age may be owing to the progressive digestion of OT and strengthening of the gastrointestinal tract barrier. Similarly, a gradual decline in plasma OT concentrations during development appears to be caused by establishment of the intestinal barrier and intestinal digestion (Ref. 49 and Fig. 3d). Digestion is at a low level at birth and increases progressively during the first week in mice and humans^49^. However, digestion of OT takes more than 30 min and is much longer in mucosal tissues^17, 50^. The increase in plasma OT within 10 min of OT administration may indicate that a relatively large amount of OT is resistant to digestion and is instead transmitted across the intestinal barrier. This result suggests that novel delivery methods for oral OT supplementation may be advantageous for the treatment of a range of conditions, such as impaired social development in preterm infants who are at risk of autism spectrum disorder, and obesity or diabetes in adults.

### Estimation of the bioavailability of OT through the intestine

An estimation of bioavailability was performed in adult mice. Fifty ng/mL of OT was detected after oral delivery of 10 μg of OT. Young adults weigh approximately 30 g and have 2.3 mL blood, so approximately 1% of oral OT can be absorbed. We have previously identified OT in the mammary tissues of mouse dams and in the milk curd inside the oesophagi of pups^13^. To determine the OT concentrations in milk, we needed to collect milk from the mothers. One common method is to collect milk after stimulating secretion with external OT. Because such milk contains external OT, we were unable to calculate the OT concentrations in milk. However, we carried out a new collection method without exogenous OT stimulation^15^. We obtained samples at 5 days after parturition, and the concentration of milk OT was 1,200 pg/mL. If we estimate a pup’s milk intake to be 100 μL, the pups will ingest 120 pg of OT. In agreement with these calculations, the increase after suckling was approximately 100 pg/mL of OT. If the total blood volume in PND5 pups is 200 μL, as calculated above, the increase is 20 pg of OT out of 120 pg, resulting in 16.6% availability. According to Tanaka *et al*.^11^, when [^3^H]OT is administered to lactating rats, the radioactivity of intact OT in the neonatal content is 4.4% of the counts obtained for the maternal plasma. These results suggest that OT in maternal blood can be transferred to milk and then to neonates. In a similar estimation, the bioavailability of 1-deamino-8-arginine vasopressin in adult humans has been examined after cutaneous, intranasal, oral, sublingual, and intrarectal administration; the bioavailability has been found to be 3.4% for intranasal, 0.1% for oral, 0% for sublingual, 0% for intrarectal, and 100% for cutaneous delivery^50^. In comparison with these results, our findings indicated a much higher level of absorption. However, it should also be noted that OT can enter the lymph and intraperitoneal space, and tight junctions and epithelial barriers are underdeveloped in all of these areas in neonates.

Application of a similar dose (10 μg) of OT to adult male mice produced higher plasma levels when administered orally (approximately 38,000 pg/mL) as compared with intestinal administration (8,000 pg/mL), as shown in Fig. 5. These results seem counterintuitive as intestinal injection close to the intestinal absorption site would seem to be more effective. However, if we assume that there are multiple absorption sites, including the buccal and oesophageal mucosa^51–53^, longer processes from oral administration could facilitate transportation under less degradation of OT. Although it has not reported in the oesophagus, OT is thought to cross the mucus–unstirred water layer in the oral cavity and then enter the circulation^54, 55^. Such buccal OT administration has been used to induce labour since 1964^56^.

In Fig. 7, the concentration of [Ile ^13^C, ^15^N]OT calculated was 27.9 ng/mL from the ratio of both peak areas and the conversion coefficient. If we take into account the dilution of the initial sample (x10), the estimated mean concentration in the blood is 346 ± 96 ng/mL (n = 4). However, this mean value is too high, even compared with values measured by mass spectrometry^47^ using deuterated OT^48^ or after a specific reduction–alkylation procedure^57^. The recovery was approximately 60%, which was calculated from total OT concentrations (600 ng in the body weight of 1. 5 g at PND3) by the injected concentration (1000 ng). This value is not far from the reported extraction efficiency and absolute recovery (above 65.8%; ref. 47). However, this raises a problem in that there is a large discrepancy in recovery between LC-MS/MS and enzyme immunoassy measurements of approximately 1%, as discussed above. Although methods to determine blood OT concentrations have not yet been established, this must be considered for such questions in the future. Nonetheless, the identical MRM peaks were scarcely detected in the plasma of RAGE KO pups at PND4–9 or after PND8 in the wild-type mice in our preliminary experiments. Our mass spectroscopy results suggest permeability as a full length form of [Ile^13^C, ^15^N]OT, with no digestion, in wild-type pups after oral administration.

### Possible mechanisms of OT permeability through the epithelial barrier

The gastrointestinal epithelium forms the boundary between the body and external environment and enables selective transportation through a permeable barrier. Transport routes through the intestinal epithelium include intracellular pathways and intercellular transport through tight junctions^34, 51, 52^. We hypothesized that RAGE functions through transcellular transportation because of its presence at the surface of villi rather than the epithelial cell body. However, we cannot ignore the possibility of rapid transportation through the paracellular pathway.

Future experiments will require *in vitro* measurements of RAGE binding to OT, including biochemical evidence of the binding of OT to RAGE with a molecular interaction tool. Such experiments are currently underway, and preliminary results show the binding affinity estimated by surface plasmon resonance to be in the micromolar range, which is lower than that of OT receptors at the subnanomolar range, thus suggesting that the OT–RAGE interaction is not identical to that between OT and its receptors.

In summary, expression of RAGE in the intestine affects OT absorption. This is the first report of a novel physiological role for RAGE beyond those in diabetes and cancer progression. Because many other ligands in addition to OT, such as AVP, can bind to RAGE, it is necessary to examine the specificity of this carrier role for OT. Instead of the inefficient nasal application of OT to healthy humans and autistic patients^58–61^, our results favour the oral administration of OT through enteric coated tablets. If feasible, this method for OT delivery may be used to treat diabetes, obesity, and autism because OT may play a role in fat loss and slowing weight gain^62, 63^.

## Methods

### Chemicals

Oxytocin (OT) and arginine vasopressin (AVP) were purchased from the Peptide Institute (Osaka, Japan). Stably labelled isoleucine [^13^C, ^15^N] OT (designated as [Ile^13^C, ^15^N]OT) was synthesized by Sigma-Aldrich Chemical Japan (Ishikari, Hokkaido, Japan). Another stably labelled OT, in which the seventh proline and eighth leucine were replaced with [^13^C, ^15^N] proline and [^13^C, ^15^N] leucine, was obtained from Scrum Co. Ltd. (Tokyo, Japan). We designated this OT isotope as [Pro&Leu^13^C, ^15^N]OT and used as an internal standard (I-STD) for the quantitation of [Ile^13^C, ^15^N]OT in mouse serum samples. LC-MS grade water, acetonitrile (ACN), formic acid, trifluroacetic acid (TFA), trichloroacetic acid (TCA), 4-kDa FITC-dextran were purchased from Wako Chemicals (Tokyo, Japan).

### Animals

*Ager* ^−/−^ mice were generated as previously described^40, 42, 64^. Briefly, we constructed a targeting vector containing two *lox*P sites flanking the neo cassette in intron 2 and another *lox*P that was inserted 0.6 kilobases (kb) 5′ upstream of exon 1 of *Ager*, which contains the translation initiation site. After gene targeting in E14-1 embryonic stem (129 background) cells, we identified six targeted clones by PCR and Southern blotting, two of which were used for further experiments. The two clones containing all three *lox*P sites in the locus gave rise to germ line chimaeras. The resultant male chimaeras were mated with female Cre-transgenic mice (CD-1 background), which transiently express Cre recombinase in eggs. Some of the newborn mice were found to carry the deleted allele that lacks both exons 1 and 2 of *Ager* and the neo cassette. For PCR genotyping, three primers were used: 5′-CCAGAGTGACAACAGAGCAGAC-3′ (primer 1), 5′-GGTCAGAACATCACAGCCCGGA-3′ (primer 2), and 5′-CCTCGCCTGTTAGTTGCCCGAC-3′ (primer 3) (nucleotides 73915–73936, 74523–74544, and 74881–74902 in GenBank accession no. AF030001, respectively).

The resultant *Ager* knockout (*Ager* ^−/−^) mice were then backcrossed to C57BL/6 mice (Charles River Japan) for more than eight generations, as described^65^. The *Ager* ^−/−^ mice were then maintained by crossing *Ager* ^−/−^ × *Ager* ^−/−^ mice for at least an additional 10 generations and then used for experiments. *Ager* ^+/−^ heterozygous mutants were obtained by crossing *Ager* ^+/−^ mice, and three genotypes of the same littermates were used in Fig. 3.

The offspring of these mice were born in our laboratory colony, weaned at 21–28 days of age and housed in same-sex groups of 3–5 animals until pairing^66^. Wild-type (C57BL/6) and *Ager* ^−/−^ mice were maintained under standard cage conditions (24 °C; 12-h light/dark cycle, lights on at 8:45 a.m.) with sawdust bedding and food and water provided *ad libitum*. Pregnant *Ager* ^−/−^ mice were transferred to clean cages 1 day before delivery. The offspring were either kept with biological or nonbiological mothers.

Virgin males and females were paired at 45 to 55 days. A single male and a single female were continuously housed together in a standard mouse maternity cage from the mating period until the delivery of pups. Each family was kept in the same box until experiments began. All family units consisting of a new sire (first-time father), dam and their first litter were experimentally naïve. All animal experiments were approved by the Committee on Animal Experimentation of Kanazawa University and performed in accordance with the Fundamental Guidelines for Proper Conduct of Animal Experiment and Related Activities in Academic Research Institutions under the jurisdiction of the Ministry of Education, Culture, Sports, Science and Technology of Japan.

### Administration of OT

Milk-borne OT was fed by mothers for 20 min after 20 min of starvation. A synthetic OT solution (100 µM; 10 μL for pups and 100 μL for adults) was administered orally. The same dose of [ILe^13^C, ^15^N]OT was delivered orally to pups. For oral administration, pups or adult mice were held by hand, and the OT solution was dispensed into their mouths using polypropylene tips.

OT (100 μM) was injected through needles (28 G) into the proximal part (1~2 cm from the pylorus) of the small intestine of pups (5 μL) and adults (100 μL) under ketamine anaesthesia (100 μg/kg intraperitoneally), after the abdominal skin was cut to expose the small intestine in the abdominal cavity. The intestine was returned to the cavity or covered with paper and rinsed with saline. For the isolated pup intestines, 30 μL of 10 μM OT was injected at the distal end.

### Blood sampling

Approximately 30 μL of blood was collected from the facial vein of neonates after quick cold anesthesia on ice (Fig. 1a; ref. 67, 68). The similar blood volume was obtained from the heart after opening of the chest of pups (Figs 1b–d, 3a,b, 6a and 7), from the carotid artery in the pup neck (Fig. 2a) and the tail vein of adults each time (Fig. 6) under ketamine anaesthesia (100 μg/kg intraperitoneally).

### Isolated intestinal preparation

Male mice were fasted for 1 h before they were euthanized by decapitation. After the intestinal cavity was opened, the gastro-oesophageal junction and the peritoneal ligaments were cut. The mesenterial tissue was stripped from the first 20 cm of the small intestine, and a 4-cm segment was excised 1~2 cm from the pylorus. The segment isolated from PND1–7 pups was washed and placed in 3 mL of incubation buffer in 24-multiwell plates at room temperature^69^. The two openings of the segment were ligated and mounted on the flat surface of the plate. Eighty percent of the segment was dipped again in a new 3 mL of incubation buffer, and the two ends were insulated by using glycerol solution. Fifty microliters of 10 μΜ OT solution was introduced into the lumen of the segment at the distal end by using a 31 G syringe needle. The incubation medium was mixed periodically by pipetting. Fifteen microliters of incubation medium was removed and stored at −80 °C for the measurement of OT concentrations.

### Enzyme immunoassay for OT and AVP

OT or AVP immunoreactivity levels were quantified by using OT and AVP enzyme immunoassay (EIA) kits (Enzo Life Sciences, NY, USA), respectively. We performed assays in tissue preparations without pre-treatment^66, 70^ to compare different tissue samples, though the fractioned sample was recommended^71, 72^. Blood samples collected as mentioned above were stored at −80 C. The blood samples were thawed and five μL of them were diluted 1:20 in assay buffer on ice and assayed. The OT or AVP assay had a sensitivity of 5 or 11 pg/mL, and the inter- and intra-assay coefficients of variation were less than 15%.

### Mass spectrometry

Standard (STD) samples of [Ile^13^C, ^15^N]OT (100 ng/mL) and [Pro&Leu^13^C, ^15^N]OT (100 ng/mL) were prepared by dilution of stock solutions with 0.1% TFA as necessary. The [Pro&Leu^13^C, ^15^N]OT (100 ng/mL) STD (10 μL) was spiked into each mouse plasma sample (10 μL) as an internal standard (I-STD), diluted with PBS (30 μL), and then de-proteinated using 50 μL of 4% (v/v) TCA. In this sample preparation, both types of OT were diluted 10-fold. The supernatant (~80 μL) was obtained after centrifugation at 14,000 rpm for 15 min at 4 °C. This sample preparation was applied to all mouse plasma samples. The standard samples were also prepared by using the control mouse plasma. Thus, the recovery of [Ile^13^C, ^15^N]OT and [Pro&Leu^13^C, ^15^N]OT were estimated be similar.

An AB Sciex 4000 QTRAP (CA, USA) was used. The prepared 25 μL sample was injected and separated by a ZORBAX 300SB-C8 column (2.1 × 150 mm, 5 μm; CA, USA) and then detected using the multiple reaction monitoring (MRM) mode of the MS/MS system. LC gradient elution was performed with two eluents, A (0.1% (v/v) formic acid/water) and B (0.1% (v/v) formic acid/acetonitrile), by linearly increasing the eluent B from 0–30% (0.0–8.0 min). The flow rate of the LC pump was set at 0.3 ml/min, and the temperature of the column was maintained at 50 °C. The MS/MS (MRM) analytical conditions used were as follows: positive ion electrospray (ESI) mode (capillary voltage 5 kV); desolvation voltage (75 V); curtain gas (nitrogen, 12 l/h, 65 psi and 600 °C); nitrogen gas pressure (65 psi), and collision voltage (30 V). The MRM transition ions and fragment types were as follows: [ILe^13^C, ^15^N]OT: (*m/z*: 1014.6 → 730.6 (b6)); and [Pro&Leu^13^C, ^15^N]OT: (*m/z*: 1021.2 → 723.2 (b6)). All data were acquired and processed with AB Sciex Analyst Version 1.4.1 (CA, USA).

### Immunohistochemistry

Sections (4 μm) of the intestine from WT and *Ager* ^−/−^ pups were stained with hematoxylin and eosin^42^. The intestine was incubated with 4′,6-diamidino-2-phenylindole (DAPI, Dojindo; Kumamoto, Japan, 1:2,000) or polyclonal rabbit anti-RAGE antibody (1:1,000; AB5484, Millipore, Billerica, MA) and washed with 0.3% Triton X-100 in PBS. They were then incubated with Alexa Fluor 488- (1:200, Invitrogen Molecular Probes).

RAGE expression in the intestine of wild-type mouse embryos (18.5 dpc) (4 μm sections from GenoStaff Inc, Tokyo, Japan) were examined using the polyclonal rabbit anti-RAGE (1:1000), Alexa Fluor 488, and DAPI. Images were taken with the EVOS FL Cell Imaging System (ThermoFisher Scientific, Yokohama, Japan).

### Statistical analysis

*P* values were calculated by using two-tailed Student’s *t*-tests for pairwise comparisons and one-way or two-way ANOVAs followed by Bonferroni’s tests for multiple comparisons, unless otherwise stated. A *P* value < 0.05 was considered to be statistically significant. Data are expressed as the mean ± SEM.

## Electronic supplementary material

Supplementary Information
