# Supplementary material for: Intestinal transepithelial permeability of oxytocin into the blood is dependent on the receptor for advanced glycation end products in mice
Source: Sci Rep. 2017 Aug 11;7:7883. doi: 10.1038/s41598-017-07949-4 (PMC5554167; doi:10.1038/s41598-017-07949-4)
Supplement: Supplementary file 1 — Supplementary Information [file 41598_2017_7949_MOESM1_ESM.pdf]

Supplementary Figures 1-3 for

Intestinal transepithelial permeability of oxytocin into the blood is dependent on the receptor for advanced glycation end products in mice

Haruhiro Higashida, Kazumi Furuhara, Agnes-Mikiko Yamauchi, Kisaburo Deguchi, Ai Harashima, Seiichi Munesue, Olga Lopatina, Maria Gerasimenko, Alla B. Salmina, Jia-Sheng Zhang, Hikari Kodama, Hironori Kuroda, Chiharu Tsuji, Satoshi Suto, Hiroshi Yamamoto, and Yasuhiko Yamamoto

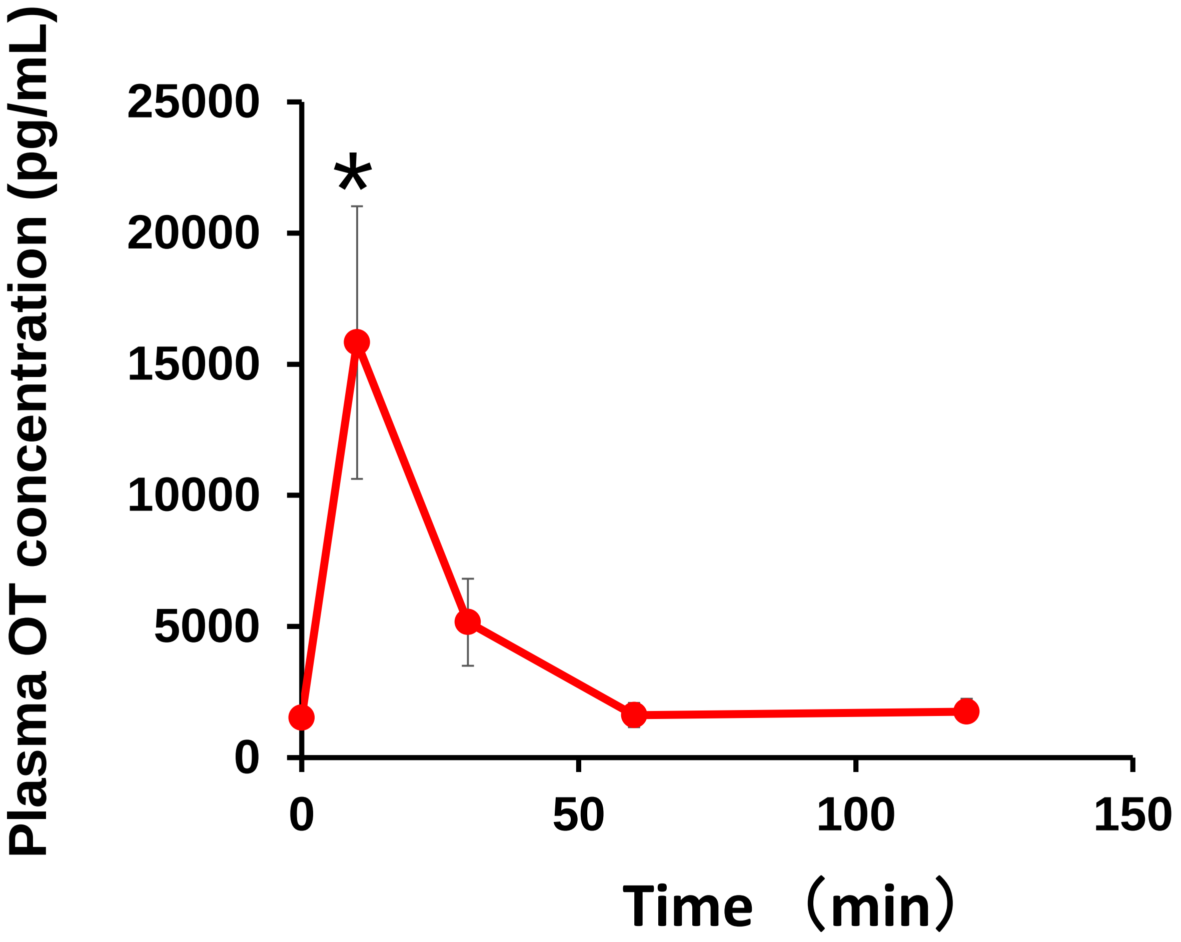

Supplementary Figure 1. Plasma OT levels in pups after intestinal delivery of OT. Blood samples were collected from the carotid artery after cutting the neck of male and female pups at PND5, 10 min after the administration of OT (100 μM x 5 μL, 0.5 μg/mouse) into the upper intestinal truct. One-way ANOVA, $F_{5,41}$ = 3.331, R^2=0.3163, P < 0.05. Bonferroni's *post hoc* tests showed significant differences, *P < 0.05 from control. N = 5-7.

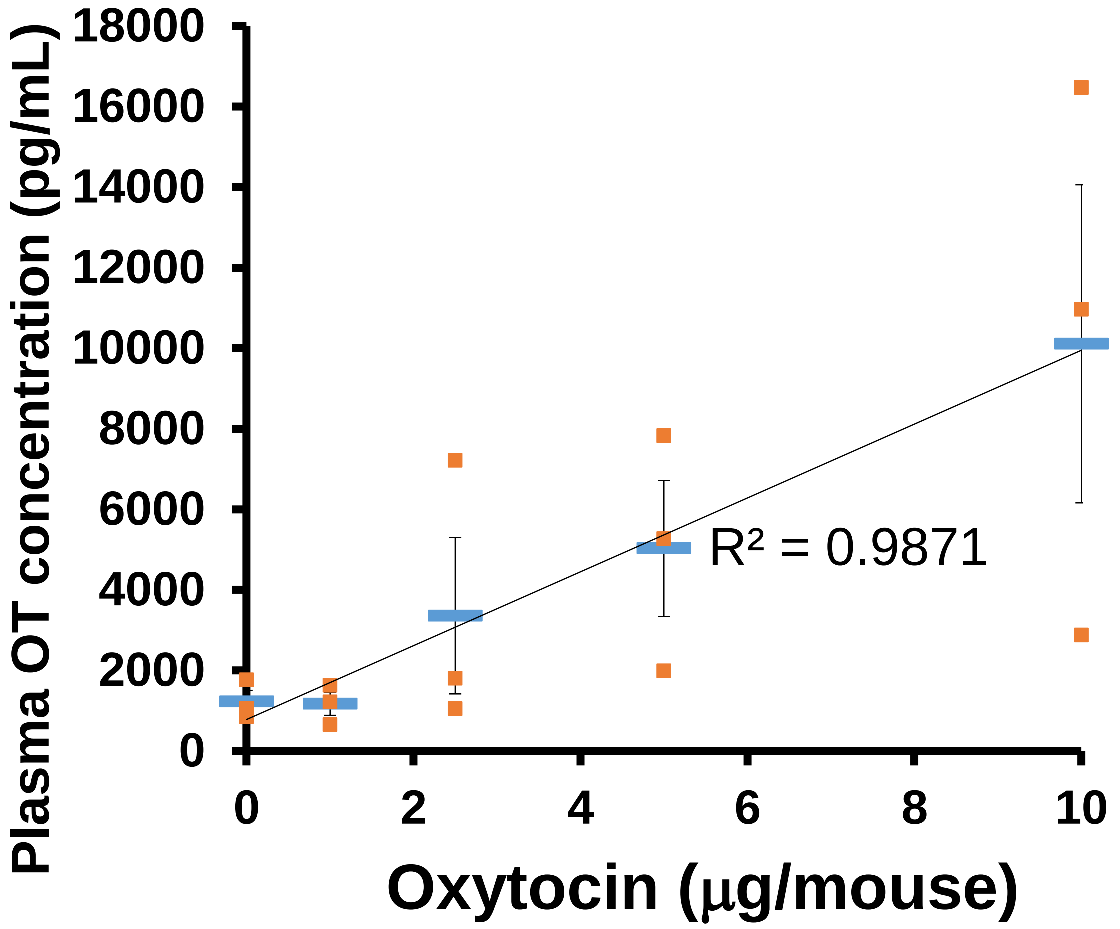

Supplementary Figure 2. Plasma OT levels in adult male mice after intestinal delivery of different concentrations of OT. Blood samples were collected from the tail 10 min after administration of different concentrations of OT into the upper intestinal tract (100 mL). N = 3.

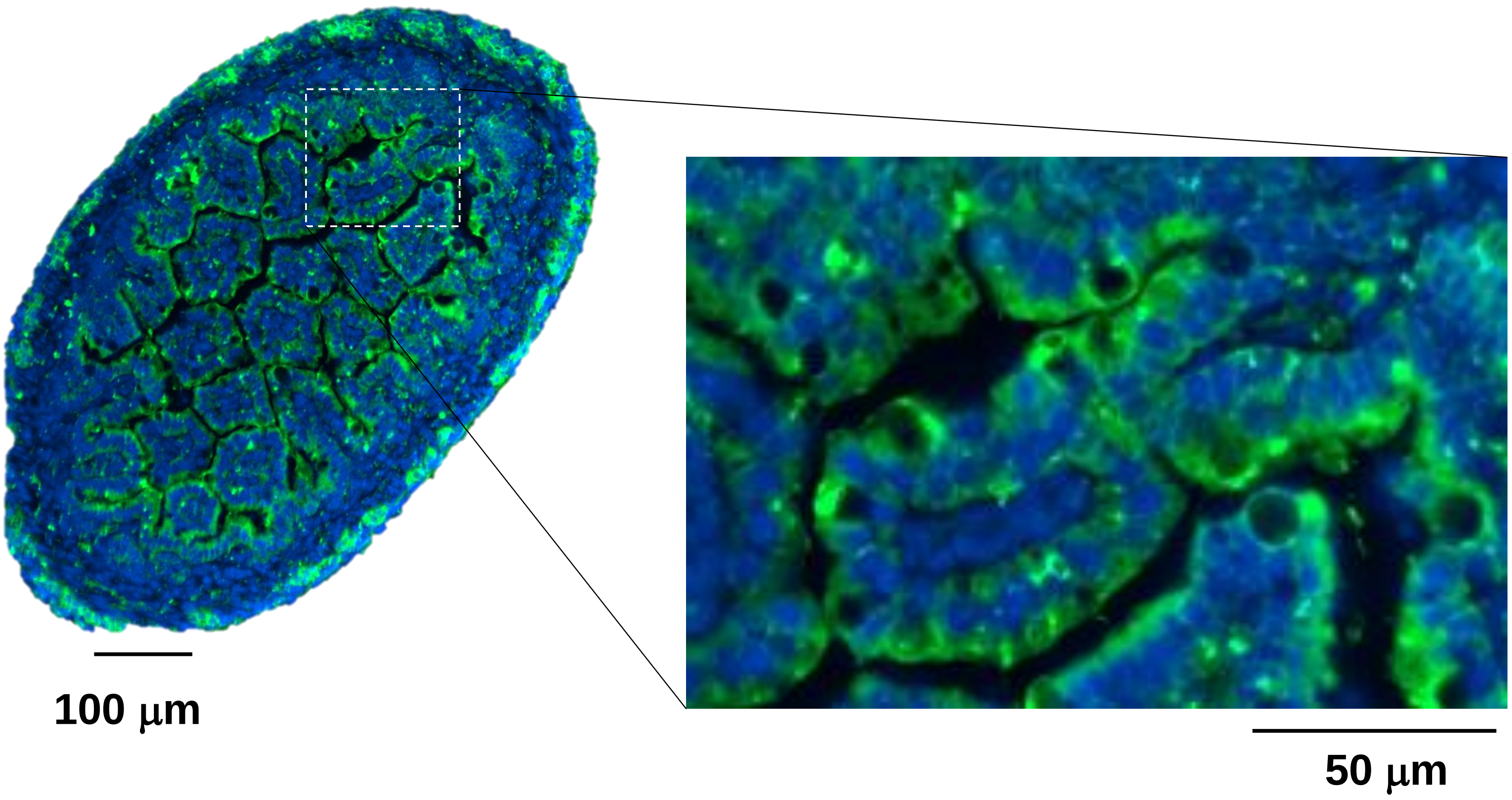

Supplementary Figure 3. Microfluorescence images. Sections of the intestines of the E18.5 embryo were immunostained with an anti-RAGE antibody (green); the nuclei were stained with DAPI. The enlarged image indicates RAGE that is present in the intestinal villi.
